# Assessment of left ventricle function in aortic stenosis: mitral annular plane systolic excursion is not inferior to speckle tracking echocardiography derived global longitudinal peak strain

**DOI:** 10.1186/1476-7120-11-45

**Published:** 2013-12-27

**Authors:** Joanna Luszczak, Maria Olszowska, Sylwia Drapisz, Wojciech Plazak, Magdalena Kaznica-Wiatr, Izabela Karch, Piotr Podolec

**Affiliations:** 1 Department of Cardiac and Vascular Diseases, Jagiellonian University Medical College, John Paul II Hospital, Pradnicka 80, 31-202 Krakow, Poland

**Keywords:** Aortic stenosis, Speckle tracking echocardiography, Global longitudinal peak strain, Mitral annular plane systolic excursion

## Abstract

**Background:**

Early detection of left ventricle (LV) systolic dysfunction is essential for management of patients with aortic stenosis (AS). Two- dimensional speckle tracking derived global longitudinal peak strain (GLPS) is more sensitive than ejection fraction (EF) but requires good image quality and is not easily accessible. The aim of the study was to compare GLPS with traditional echocardiographic parameter- mitral annular plane systolic excursion (MAPSE) in AS.

**Material and methods:**

In consecutive patients with moderate to severe AS and LV ejection fraction ≥ 50% standard echocardiography and two-dimensional speckle tracking echocardiography were performed. Mitral annular plane systolic excursion and global longitudinal peak strain were obtained from apical echocardiographic views.

**Results:**

A total of 82 patients were examined, median age was 68 (60–78), 56% of them were men. There was a positive correlation between aortic valve area index (AVAI) and: MAPSE (r = 0.334, p = 0.002), MAPSE indexed for body surface area- MAPSEI (r = 0.349, p = 0.001) and GLPS (r = 0.342, p = 0.002) but not EF (r = 0.031, p = 0.782). A positive correlation was found between GLPS and MAPSE (r = 0.558, p < 0.001) and between GLPS and MAPSEI (r = 0.543, p < 0.001). All above parameters were significantly lower in symptomatic patients compared to asymptomatic subjects (GLPS: -13.82 ± 3.56 vs. -16.39 ± 3.16%, p = 0.002, MAPSE: 10.49 ± 1.91 vs. 11.95 ± 1.82 mm, p = 0.001 and MAPSEI: 5.66 (4.83-6.6) vs. 6.46 ± 0.97 mm/m^2^, p = 0.005).

**Conclusion:**

Despite the development of the modern echocardiographic techniques, mitral annular plane systolic excursion can still be used as a sensitive tool to detect early longitudinal LV systolic dysfunction.

## Introduction

Aortic stenosis (AS) is the most common type of valvular heart disease [1]. Early detection of left ventricle (LV) systolic function impairment is essential for the management of patients with AS. LV ejection fraction (EF) below 50% is associated with worse outcome after aortic valve replacement [2] and consist an indication to the surgical treatment even in asymptomatic patients [3]. However, EF remains within normal values for a long period of time, despite progressive deterioration of the LV long-axis contractility [4-10]. Two-dimensional speckle tracking echocardiography (2D-STE) is an angle independent method that enables offline measurements of the longitudinal, radial and circumferential strain and strain rate [11-13]. 2D-STE derived global longitudinal peak strain (GLPS) is an relatively easy to obtain single parameter quantifying LV long-axis systolic function. It has been shown that GLPS is progressively affected with increasing AS severity [4-6]. Furthermore, as we have previously demonstrated, it is even more reduced in symptomatic patients in comparison to asymptomatic subjects [8]. However, speckle tracking echocardiography requires good imaging quality and dedicated software that is not accessible in every echocardiographic laboratory. Thus, we aimed to compare usefulness of the more traditional and easy-available methods in detecting early LV systolic dysfunction. Mitral annular plane systolic excursion (MAPSE) is a parameter that quantifies mitral annulus plain displacement during systole, reflecting the global LV long-axis systolic function [14]. It has been demonstrated that MAPSE is decreased in AS and declines with the increasing AS severity [15,16]. MAPSE is less dependent of the image quality as the mitral annulus is usually highly echogenic. The study was undertaken to compare GLPS with mitral annular plane systolic excursion.

## Materials and methods

Consecutive patients with moderate to severe AS and preserved LV systolic function (LVEF ≥ 50%), diagnosed in Cardiac and Vascular Diseases Department, Jagiellonian Unievestity Medical College, Krakow, Poland were enrolled. All patients underwent physical examination, standard blood analysis and transthoracic echocardiography. Detailed medical histories were taken, including the presence of AS symptoms (chest pain, dyspnea, syncope). Exclusion criteria included: unstable coronary artery disease (CAD), LVEF < 50%, regional LV wall motion abnormalities, concomitant moderate or severe disease of another valve, mitral annular calcification, atrial fibrillation and severe renal or hepatic insufficiency.

The study was approved by the local ethics committee of the Jagiellonian University and written informed consent was obtained from all the participants.

### Echocardiography

**Figure 1:**
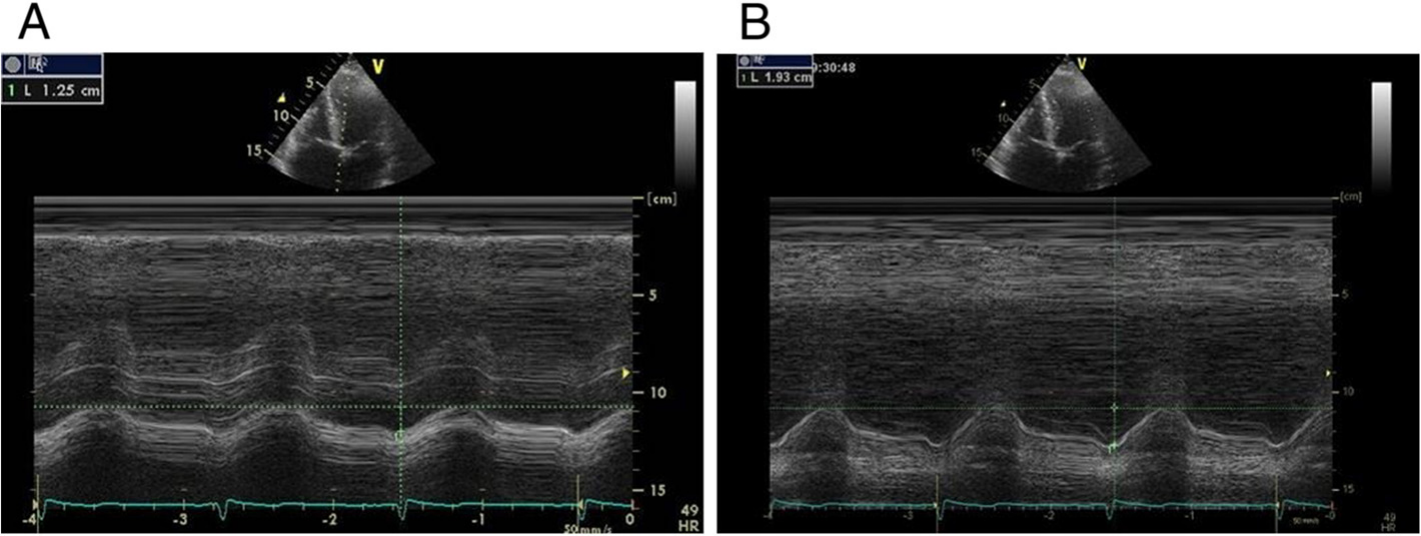
**Measurement of the mitral annular plane systolic excursion (MAPSE).** MAPSE was measured at the septal side **(A)** and the lateral side **(B)** of the atrioventricular plane by M-Mode and the average value was calculated.

**Figure 2:**
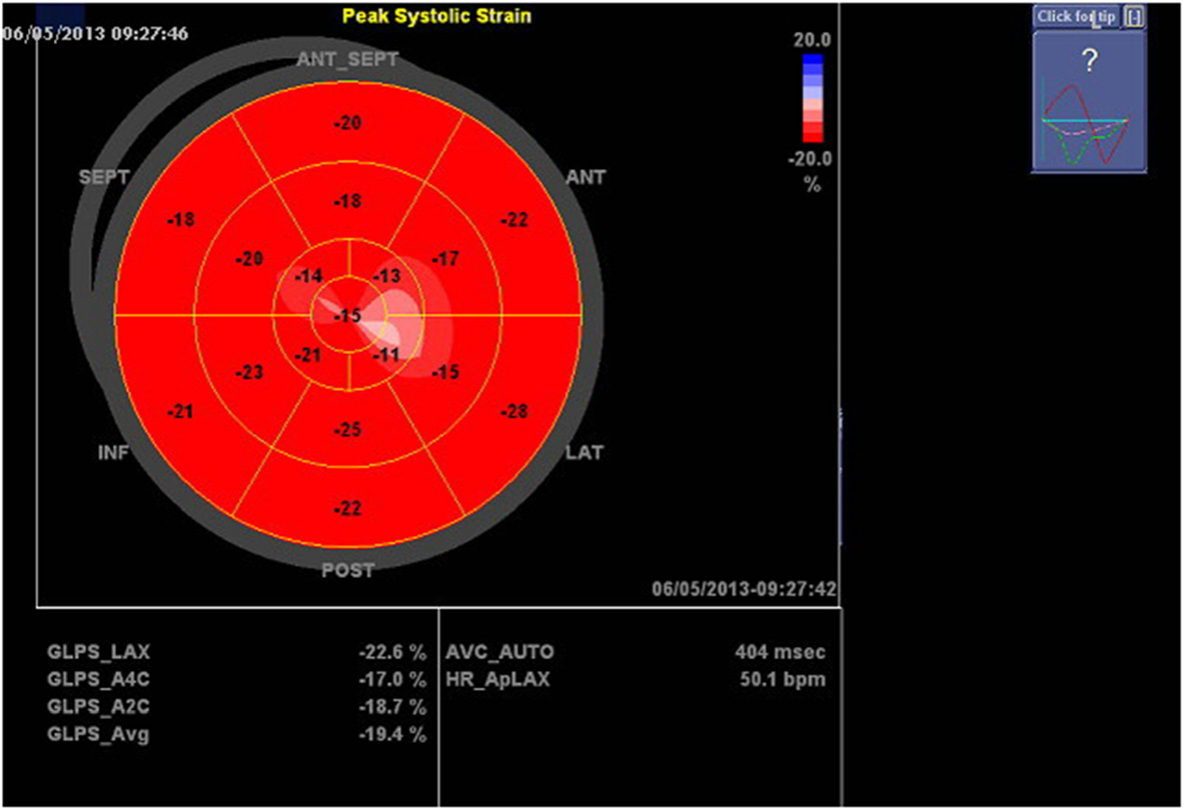
**Global and segmental longitudinal strain.** The “bull-eye” presentation shows the longitudinal strain in 17 segments of the left ventricle and calculated global longitudinal peak strain (GLPS_Avg).

Transthoracic echocardiography was performed using Vivid Seven GE Medical Systems equipment (Horton, Norway) and digitally stored for further analysis on the EchoPAC version 108.1.12 (GE Healtcare). Measurements were undertaken in the left lateral decubitus position. LV end-systolic volume (LVESV), end-diastolic volume (LVEDV) and ejection fraction were measured by the modified bi-plane Simpson rule, LV mass were calculated using ASE formula [17]. The mean and the peak transaortic gradients were measured by continuous wave Doppler method. Aortic valve area (AVA) was calculated using the continuity method [18]. AVA, LV mass, LVESV and LVEDV and MAPSE were indexed for the body surface area (BSA). Mitral annular plane systolic excursion was measured using M-mode imaging in the apical four-chamber view. The M-mode cursor was placed on the septal and lateral mitral annulus as much parallelly as possible to the LV walls (Figure 1), then both values were averaged. Global longitudinal peak strain was measured using speckle tracking echocardiography as previously described [8]. The ECG-gated images were obtained in apical long-axis, four- and two- chamber views at the frame rate of 50–70 per second and stored digitally. The endocardial border of the myocardium was traced automatically and corrected manually, if needed. Segmental strain was presented as a bull- eye map and GLPS was automatically calculated (Figure 2). Early diastolic mitral annular velocity was measured at the lateral site of the mitral annulus by pulsed wave tissue Doppler echocardiography (TDE). AS was considered as severe for AVA < 1 cm^2^ or AVA index (AVAI) < 0.6 cm^2^/m^2^.

### Statistical analysis

Statistical analysis was performed using the STATISTICA 10.0 software. The Shapiro-Wilk test was used to determine normal distribution. Levene’s test was used to determine the homogeneity of variance. Continuous variables are shown as mean ± standard deviation (SD) or median and interquartile range (IQR). Categorical variables are presented as the number of patients and percentage. Comparisons between the groups were made by t-test for normally distributed continuous variables, Mann- Whitney U test for non-normally distributed continuous variables and χ^2^ test for categorical variables. The Pearson’s or Spearman’s correlation was used to examine linear correlation between the numerical variables. Receiver operating characteristics curve (ROC) analysis with area under the curve (AUC) calculation was used for the determination of cut-off values of MAPSE and GLPS o predict clinical symptoms. The value of p < 0.05 was considered statistically significant.

## Results

### Demographic, clinical and echocardiographic characteristics

A total of 82 patients were studied. Of these, 55 patients (67%) had severe AS. The median age was 68 (60–78) years, 46 (56%) of them were men. Patients were divided into the two subgroups according to their symptomatic status: 53 patients (65%) were symptomatic and 29 (35%) were asymptomatic. Basic demographic and clinical data of the patients are summarized in Table 1.

**Table 1:** Demographic and clinical characteristics of the patients with aortic stenosis (AS)

	**Total group (n=82)**	**Severe aortic stenosis (n=55)**	**Moderate aortic stenosis (n=27)**	p-value*
Age (years)	68 (60-78)	70 (61-79)	68 (58-78)	0.399
Males, n (%)	46 (56)	30 (54.6)	16 (59.3)	0.686
Body surface area (m²)	1.85 ± 0.2	1.85 ± 0.21	1.86 ± 0.17	0.849
Body mass index (kg/ m²)	26.6 ± 3.6	26.3 (23.7-28.3)	26.8 ± 3.1	0.456
Smoking, n (%)	15 (18)	11 (20)	4 (14.8)	0.568
Symptomatic, n (%)	53 (65)	41 (74.6)	12 (44.4)	0.007
Hypertension, n (%)	61 (74)	40 (72.7)	21 (77.8)	0.622
Diabetes, n (%)	19 (23)	13 (23.6)	6 (22.2)	0.887
Coronary artery disease, n (%)	36 (44)	25 (45.5)	11 (40.7)	0.686

*between severe and moderate AS.

In the whole group median AVAI was of 0.45 (0.36-0.62) cm^2^/m^2^, with the mean ejection fraction of 63 ± 7%. By definition, patients with severe AS had higher transaortic gradients and lower AVA and AVAI. They also have significantly higher left ventricle end-systolic volume index and lower GLPS, MAPSE and MAPSE index (MAPSEI) compared to moderate AS patients. The echocardiographic characteristics of the study population is presented in Table 2.

**Table 2:** Echocardiographic parameters in patients with aortic stenosis

	**Total group (n=82)**	**Severe aortic stenosis (n=55)**	**Moderate aortic stenosis (n=27)**	p-value*
Ejection fraction -EF (%)	63 ± 7	63.5 ± 7.2	61.7 ± 6.5	0.272
Left ventricle end-diastolic volume index (ml/m²)	65.6 ± 23.8	62 ± 23.1	72.8 ± 23.9	0.053
Left ventricle end-systolic volume index (ml/m²)	26.6 (17.2-38.1)	23.5 (15.5-31.4)	33.4 ± 13	0.007
Left ventricle mass index (g/m²)	161 ± 47.8	165.7 ± 45.9	151.5 ± 50.8	0.215
Left atrial volume index (ml/m²)	38 ± 14.5	39 ± 15.3	35.9 ± 12.7	0.369
Peak aortic gradient (mmHg)	66.5 (52-96)	80 (65-111)	48 ± 16	<0.001
Mean aortic gradient (mmHg)	43 (33-57)	50 (42-71)	28 ± 11	<0.001
Aortic valve area- AVA (cm²)	0.85 (0.65-1.14)	0.72 ± 0.18	1.3 ± 0.2	<0.001
Aortic valve area index- AVAI (cm²/m²)	0.45 (0.36-0.62)	0.39 (0.32-0.45)	0.67 (0.62-0.76)	<0.001
Global longitudinal peak strain - GLPS (-%)	14.73 ± 3.62	14.17 ± 3.59	15.87 ± 3.47	0.045
Mitral annular plain systolic excursion - MAPSE (mm)	11 ± 2	10.64 ± 1.95	11.76 ± 1.9	0.016
MAPSE index (mm/m²)	6 ± 1.1	5.79 ± 1.01	6.39 ± 1.2	0.02
E/E’ ratio	9.8 (7-14.4)	10.6 (7.1-14.3)	8.7 (6.9-14.5)	0.386
Early diastolic mitral annular velocity- E’ (cm/s)	6 (5-9)	6.6 ± 2.5	7.7 ± 3.1	0.1

*between severe and moderate AS.

### Correlations between assessed parameters quantifying longitudinal LV systolic function and aortic stenosis severity

**Figure 3:**
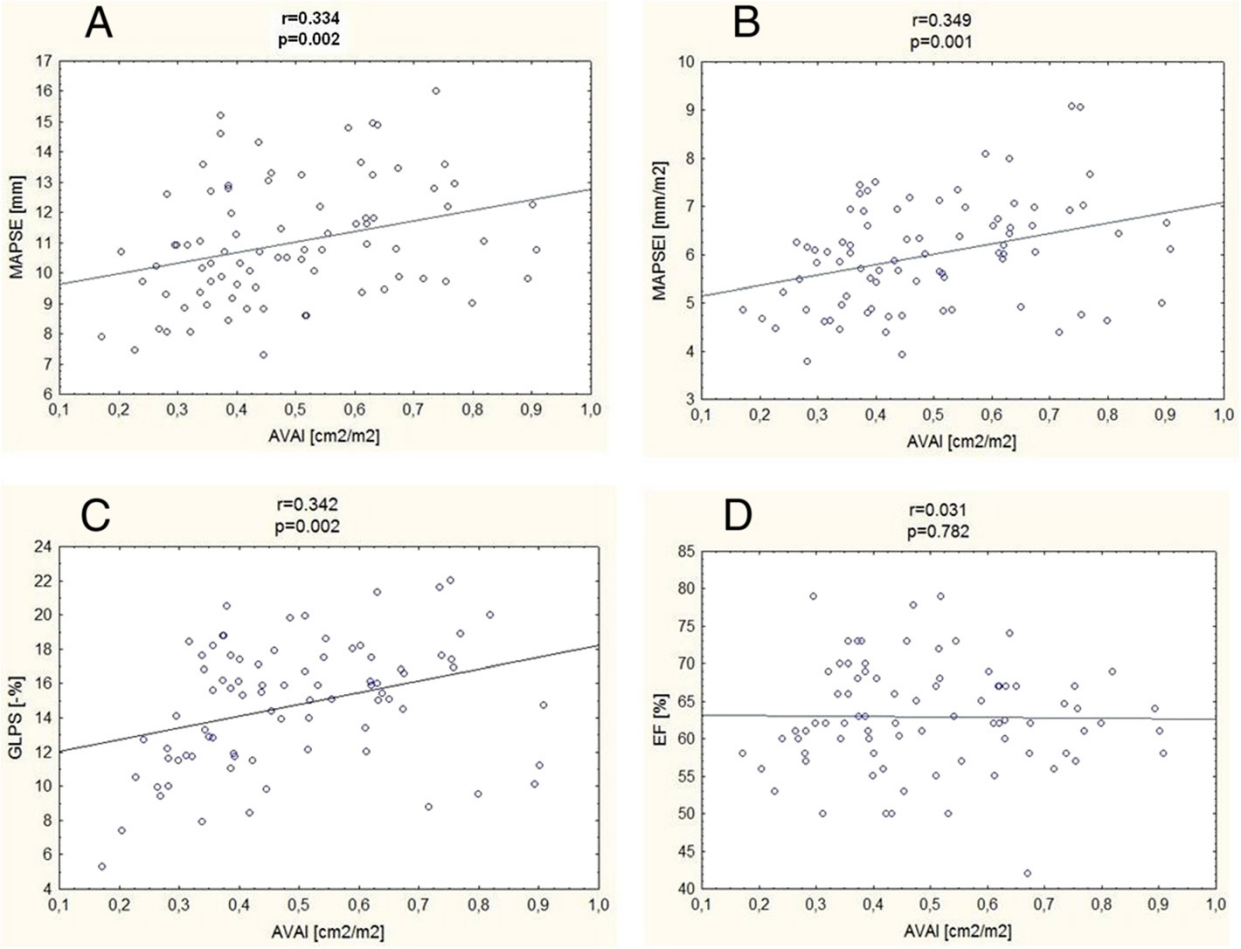
**Correlations between aortic valve area index (AVAI) and parameters assessing left ventricle systolic function.** MAPSE- mitral annular plane systolic excursion **(A)**, MAPSEI- mitral annular plane systolic excursion index **(B)**, GLPS- global longitudinal peak strain **(C)**, EF- ejection fraction **(D)**.

There was a positive correlation between AVAI and: MAPSE (r = 0.334, p = 0.002), MAPSEI (r = 0.349, p = 0.001) and GLPS (r = 0.342, p = 0.002), but not with EF (r = 0.031, p = 0.782), as presented in Figure 3.

EF correlated weakly with GLPS (r = 0.233, p = 0.039) and MAPSEI (r = 0.278, p = 0.011) but not with MAPSE (r = 0.174, p = 0.118).

**Figure 4:**
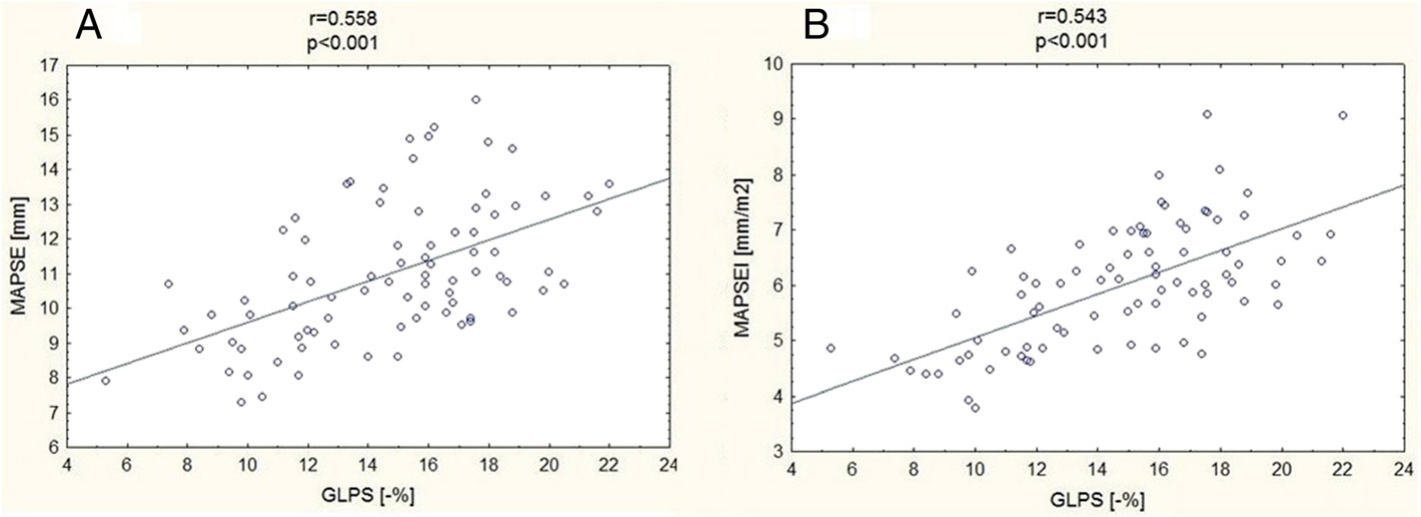
**Correlations between global longitudinal peak strain (GLPS) and M-Mode derived parameters.** MAPSE- mitral annular plane systolic excursion **(A)**, MAPSEI- mitral annular plane systolic excursion index **(B)**.

A good positive correlation was found between GLPS and MAPSE (r = 0.558, p < 0.001) and between GLPS and MAPSEI (r = 0.543, p < 0.001), what is shown in Figure 4.

### Comparison between symptomatic and asymptomatic subgroups of the patients

Patients with symptoms were significantly older than those asymptomatic while other demographic and clinical data such as prevalence of hypertension, diabetes or coronary artery disease were comparable between these subgroups (Table 3).

**Table 3:** Demographic and clinical characteristics of the patients with asymptomatic and symptomatic aortic stenosis

	**Asymptomatic aortic stenosis (n=29)**	**Symptomatic aortic stenosis (n=53)**	**p**
Age (years)	66 (49-73)	70 ± 11	0.015
Males, n (%)	15 (51.7)	31 (58.5)	0.555
Body surface area (m²)	1.86 ± 0.19	1.85 ± 0.2	0.797
Body mass index (kg/ m²)	26 ± 2.8	26.9 ± 4	0.315
Smoking, n (%)	7 (24.1)	8 (15.1)	0.311
Hypertension, n (%)	18 (62.1)	43 (81.1)	0.059
Diabetes, n (%)	5 (17.2)	14 (26.4)	0.347
Coronary artery disease, n (%)	10 (34.5)	26 (49)	0.204

However, symptomatic patients had significantly higher transvalvular peak and mean pressure gradients and a lower AVAI compared to asymptomatic subjects. Furthermore, all parameters quantifying longitudinal LV function (GLPS, MAPSE and MAPSEI) were significantly lower in symptomatic subgroup, as presented in Table 4. Also E’ velocity (but not E/E’ ratio) was significantly lower in patients with symptoms. Left ventricle ejection fraction, LV volumes and masses indexed for BSA did not differ between symptomatic and asymptomatic patients.

**Table 4:** Echocardiographic parameters in the patients with asymptomatic and symptomatic aortic stenosis

	**Asymptomatic aortic stenosis (n=29)**	**Symptomatic aortic stenosis (n=53)**	**p-value**
Ejection fraction –EF (%)	63.4 ± 5.4	62.7 ± 7.7	0.643
Left ventricle end-diastolic volume index – LVEDVI (ml/ m²)	55.8 (44.1-80)	67.4 ± 24.7	0.364
Left ventricle end-systolic volume index –LVESVI (ml/ m²)	26.5 (19.1-33.5)	28.1 ± 12.9	0.84
Left ventricular mass index (g/m²)	146 (121-173)	168 ± 46	0.08
Left atrial volume index –LAVI (ml/ m²)	35.4 ± 13.9	39.3 ± 14.7	0.252
Peak aortic gradient (mmHg)	57 (40-67)	80 ± 30	0.014
Mean aortic gradient (mmHg)	33 (23-44)	46 (40-60)	0.005
Aortic valve area – AVA(cm²)	1.07 ± 0.3	0.74 (0.6-0.92)	<0.001
Aortic valve area index –AVAI (cm²/m²)	0.58 ± 0.17	0.4 (0.34-0.52)	<0.001
Global longitudinal peak strain- GLPS (-%)	16.39 ± 3.16	13.82 ± 3.56	0.002
Mitral annular plain systolic excursion– MAPSE (mm)	11.95 ± 1.82	10.49 ± 1.91	0.001
MAPSE index (mm/m²)	6.46 ± 0.97	5.66 (4.83-6.6)	0.005
E/E’ ratio	0.68 (0.49-0.98)	0.65 (0.51-0.85)	0.17
Early diastolic mitral annular velocity- E’ (cm/s)	8.07 ± 2.69	6 (4-8)	0.007

### Receiver operating characteristic (ROC) curves for clinical symptoms

**Figure 5:**
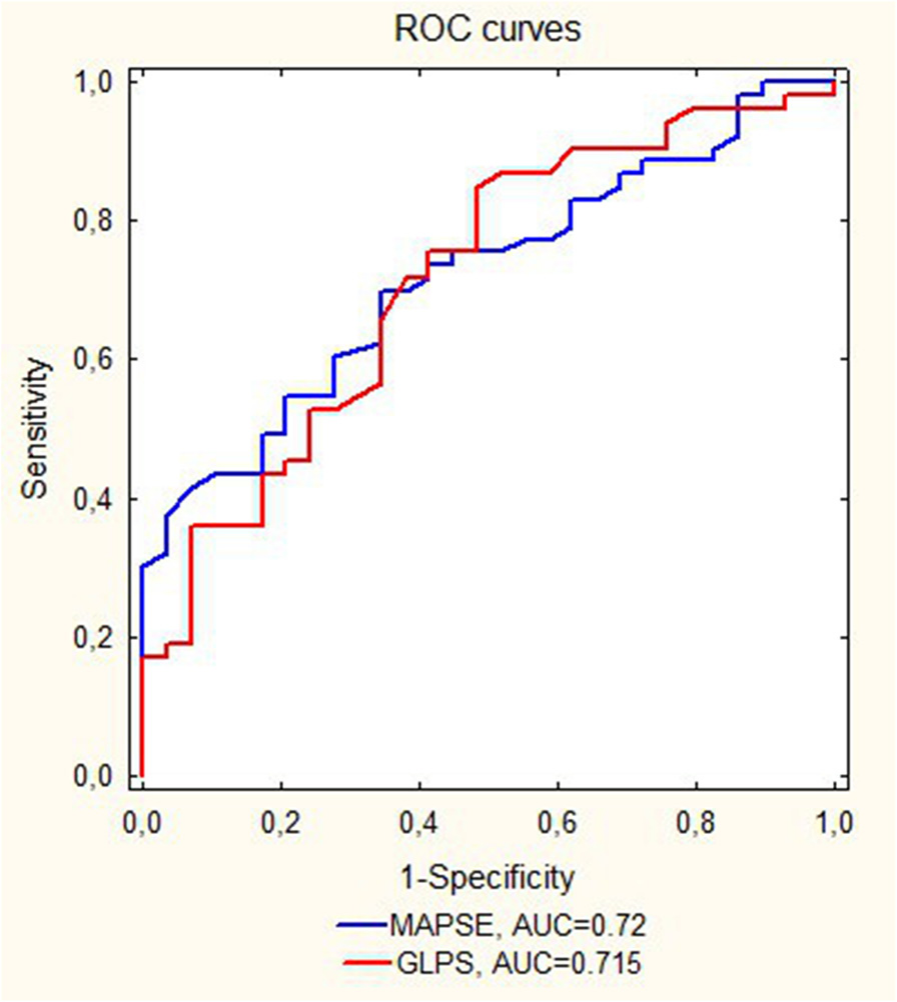
**Receiver operating characteristic (ROC) curves for the prediction of symptoms.** MAPSE- mitral annular plane systolic excursion, GLPS- global longitudinal peak strain, AUC- area under the curve.

Both GLPS and MAPSE had similar area under the curve (AUC) for symptoms prediction, p = 0.933, as shown in Figure 5. The cut-off value for GLPS was −17.1% with the sensitivity 84.9% and the specificity 51.7%, and for MAPSE 10.9 mm with the sensitivity 69.8% and the specificity 65.5%.

## Discussion

In the present study, we show that 2D speckle tracking derived global longitudinal peak strain, mitral annular peak systolic excursion, as well as mitral annular peak systolic excursion index correlate inversely with the severity of AS. Moreover, there was a good correlation between GLPS and MAPSE and MAPSEI. All of these parameters quantify long-axis LV systolic function.

Although more than 50% of the stroke volume is generated by the longitudinal LV contractility [19,20], ejection fraction may remain preserved even when LV long- axis systolic function is impaired [4-10]. This is possible due to the changes in LV geometry, with increased LV wall thickness and decreased LV radius as a compensatory mechanism minimizing the negative influence of the high afterload on the LV stroke volume. It was demonstrated that, impairment of the longitudinal contractility in AS precedes the deterioration of the radial and circumferential function of LV [9,21,22]. Furthermore, Cramariuc et al. [23] showed that average longitudinal strain depends on the LV geometry and as a marker of LV function is of the lowest value in concentric hypertrophy.

### Speckle tracking echocardiography and mitral annular plane displacement

Speckle tracking echocardiography has been described as a method assessing LV multidirectional function. This technique enables to quantify global and regional LV contractility [11-13]. Many studies reported decreased longitudinal strain measured in 2D-STE technique in patients with aortic stenosis in spite of normal EF [4-8,24-26]. It has been also revealed that patients with lower GLPS before aortic valve replacement may have worse prognosis after surgery [27]. However, the similar observation have been obtained previously with regard to MAPSE [15,16,24]. Vinereanu et al. [28] have shown that global LV function can be estimated by mitral annular excursion. They have found correlations between LV ejection fraction and mitral annular plane systolic motion, that were more pronounced in subjects without regional LV motion abnormalities caused by previous myocardial infarction. Similar relationships were noticed in patients with heart failure with preserved ejection fraction: Wenzelburger et al. [29] have shown that MAPSE correlates with longitudinal strain at rest end during exercise.

Currently, in the era of the development of more advanced techniques, the measurement of the atrioventricular plane displacement may seem to be unnecessary. However, it may be of great value in many cases. MAPSE does not require good acoustic window, thus can by applied when visualization of the endocardial borders is not obtainable. Moreover, this old technique can be performed at the bedside. In the study of Bergenzaun et al. [30], performed on critically ill patients with sepsis, MAPSE was obtainable in all the patients with low inter- and intra-observer variability (4.4% and 5.3%, respectively). The authors also showed that MAPSE was an independent predictor of a 28-day mortality. Another advantage of M-mode measurements is the possibility of assessing MAPSE during exercise test when the target heart rate (HR) is high, especially in young individuals who have predicted maximal values of HR close to 200 beats per minute [31]. By contrast, images for 2D-STE are obtained with low frame rate, thus in higher heart rates the analysis may not be possible [32].

While there are no standards concerning the point where MAPSE should be measured, we decided to use the average value from septum and lateral wall.

The data concerning the relationship between 2D-STE derived global longitudinal peak strain and mitral annular plane excursion in aortic stenosis are limited. GLPS is a parameter calculated from all 17 LV segments while MAPSE in the present study was assessed in the LV lateral wall and interventricular septum only. Thus, the measurement of MAPSE is more simple and does not require good image quality of all LV segments- only the mitral annulus, being the highly accessible and sensitive method of early LV function impairment in aortic stenosis. Of note, in the current study, all LV function parameters (GLPS, MAPSE and MAPSEI) were decreased in symptomatic patients, while there was no difference in ejection fraction between these subgroups, what may have the prognostic clinical value.

Furthermore, both GLPS and MAPSE were characterized by having the AUC for symptoms prediction exceeding 0.7, and that could differentiate symptomatic from asymptomatic patients. Similar observation with regard to GLPS were obtain by Laffite et al. [9] with the cut-off value for global longitudinal strain of −18% (with sensitivity 68% and specificity 75%, AUC = 0.77). The slightly higher threshold than that in the our study can be explained by the methodological differences: in their study symptoms were induced by the exercise test, while during taking the history patients denied symptoms. A cut-off value for MAPSE for predicting symptoms was previously not well established.

### Limitations of the study

The main limitation of this study is heterogeneity of the study group. The concomitant diseases may impinge on the left ventricle longitudinal contractility. Nevertheless, we have decided to include patients with hypertension, diabetes and coronary artery disease because those diseases are common in the population of patients with aortic stenosis.

MAPSE may usually be normalized to the heart size. However, in this study, we indexed MAPSE to the body surface area, as we did with the other values: aortic valve area, left ventricle mass and volumes.

## Conclusions

The present study suggest that, in spite of the development of the modern echocardiographic technologies, mitral annular plain systolic excursion can still be used to asses LV systolic longitudinal function. The advantage of this older technique may particularly be seen in suboptimal acoustic windows or when the dedicated software for speckle tracking echocardiography is not available.

## Competing interests

The authors declare that they have no competing interests.

## Authors’ contributions

JL designed the study, collected the data, interpreted the data and drafted the article. MO participated in the design of the study and helped to draft the manuscript. SD collected the data and interpreted the data. WP interpreted the data and helped to draft the manuscript. MKW and IK collected the data. PP interpreted the data. All authors read and approved the final manuscript.
